# Supplementary material for: Assessment of a Takagi–Sugeno-Kang fuzzy model assembly for examination of polyphasic loglinear allometry
Source: PeerJ. 2020 Jan 6;8:e8173. doi: 10.7717/peerj.8173 (PMC6951296; doi:10.7717/peerj.8173)
Supplement: Supplemental Information 1 — We provide the Echavarria Heras et al 2018, (Mascaro et al., 2011) AND hUXLEY 1932 data sets. For (De Robertis & Williams, 2008) data please contact Alex De Robertis: alex.derobertis@noaa.gov [file peerj-08-8173-s001.zip › DATA/De Robertis and Williams 2008 Raw Data.docx]

De Robertis and Williams 2008 data

We acquired the De Robertis and Williams 2008 raw data directly from the authors. We agreed using the data solely for fitting the TSK fuzzy model. We were not allowed to release the data to third parties. Nevertheless for accessing the data for reviewing please contact Professor De Robertis : [alex.derobertis@noaa.gob](mailto:alex.derobertis@noaa.gob)
