# Supplementary figures and images for: Assessment of a Takagi–Sugeno-Kang fuzzy model assembly for examination of polyphasic loglinear allometry
Source: PeerJ. 2020 Jan 6;8:e8173. doi: 10.7717/peerj.8173 (PMC6951296; doi:10.7717/peerj.8173)

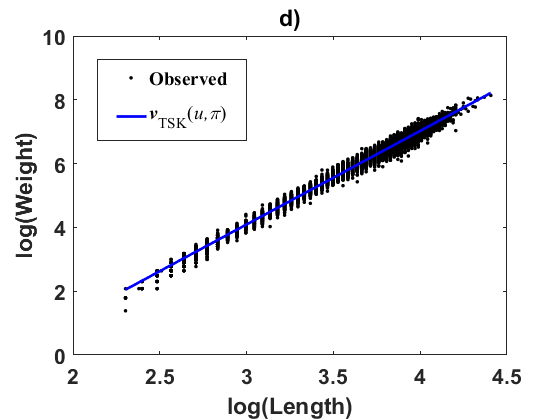

Supplement: Supplemental Information 2 [file peerj-08-8173-s002.zip › METHODS/PLA/Robertis/HOMOSCEDASTIC/Fig_11d.png]
